# Mutation of histone H3 serine 86 disrupts GATA factor Ams2 expression and precise chromosome segregation in fission yeast

**DOI:** 10.1038/srep14064

**Published:** 2015-09-15

**Authors:** Kim Kiat Lim, Terenze Yao Rui Ong, Yue Rong Tan, Eugene Guorong Yang, Bingbing Ren, Kwi Shan Seah, Zhe Yang, Tsu Soo Tan, Brian W. Dymock, Ee Sin Chen

**Affiliations:** 1 Department of Biochemistry, School of Medicine, National University of Singapore, Singapore; 2 National University Health System (NUHS), National University of Singapore, Singapore; 3 Synthetic Biology Research Consortium, National University of Singapore, Singapore; 4 NUS Graduate School for Integrative Sciences and Engineering, National University of Singapore, Singapore; 5 Department of Pharmacy, Faculty of Science, National University of Singapore, Singapore; 6 School of Chemical & Life Sciences, Nanyang Polytechnic, Singapore

## Abstract

Eukaryotic genomes are packed into discrete units, referred to as nucleosomes, by organizing around scaffolding histone proteins. The interplay between these histones and the DNA can dynamically regulate the function of the chromosomal domain. Here, we interrogated the function of a pair of juxtaposing serine residues (S86 and S87) that reside within the histone fold of histone H3. We show that fission yeast cells expressing a mutant histone H3 disrupted at S86 and S87 (*hht2-S86AS87A*) exhibited unequal chromosome segregation, disrupted transcriptional silencing of centromeric chromatin, and reduced expression of Ams2, a GATA-factor that regulates localization of the centromere-specific histone H3 variant CENP-A. We found that overexpression of *ams2*^+^ could suppress the chromosome missegregation phenotype that arose in the *hht2-S86AS87A* mutant. We further demonstrate that centromeric localization of SpCENP-A^*cnp1-1*^ was significantly compromised in *hht2-S86AS87A*, suggesting synergism between histone H3 and the centromere-targeting domain of SpCENP-A. Taken together, our work presents evidence for an uncharacterized serine residue in fission yeast histone H3 that affects centromeric integrity via regulating the expression of the SpCENP-A-localizing Ams2 protein. [173/200 words]

In the nuclear compartment, eukaryotic DNA is wrapped around structural proteins known as histones to create a higher-ordered, packaged chromatin structure^1^. The most fundamental subunit of chromatin is the nucleosome, which is composed of approximately 146 base pairs of DNA making 1.6 turns around 8 histone molecules—two copies each of histone H3, H4, H2A and H2B. Histone proteins share a universal primary architecture consisting of a structured histone fold domain embedded within the nucleosome core and joined at both ends by unstructured tail regions that protrude into the exterior of the nucleosomal core through DNA gyres^1^,^2^.

Residues on histone proteins are heavily decorated with post-translational modifications (PTMs). These PTMs function to control chromatin compaction and/or to act as docking sites for other chromatin-modifying factors that host PTM-binding motifs^1^,^3^,^4^. These epigenetic mechanisms are particularly well characterized for PTMs planted on the N-terminal domain (NTD) of histone H3, which ensure the fidelity of cellular processes important in the maintenance of genomic integrity, such as chromosome segregation, DNA damage repair, DNA replication and transcription^5^,^6^,^7^,^8^. Phosphorylation is one such PTM on histone H3 that was, until recently, rather unstudied. New mechanistic insights have elucidated roles for several phosphorylation sites on the histone H3 NTD: phosphorylation at histone H3 Thr 3 (H3T3) recruits the Aurora kinase-containing chromosome passenger complex^9^,^10^,^11^; H3T6 phosphorylation regulates the accessibility of the H3 Lys 4 (H3K4) demethylase LSD1 to its target^12^; H3T11 phosphorylation directs the localization of the H3K4 methylating Set1/MLL complex^13^; and phosphorylation of H3 Tyr 41 (H3Y41) counteracts the binding of a human HP1 protein in leukemic cells^14^. Furthermore, phosphorylation at H3 Ser 10 (H3S10) and H3S28 is cell cycle-regulated (peaking in the M-phase) and these phosphorylated residues act as interaction sites for the 14-3-3 protein^15^.

Compared with the histone H3 NTD, much less is known about the phosphorylation events of the histone fold domains. Even though there exist many residues that could be potentially phosphorylated—threonine, serine and tyrosine—only a few sites have been shown to be phosphorylated, with fewer functionally characterized. Those that have been studied include H3T45, which has been linked to apoptosis^16^; H3T118, which has been demonstrated to increase chromatin accessibility and mobility *in vitro*, and hence implicated in promoting chromatin remodelling^17^; H3T80, which has been identified very recently in mitotic-phase cells and is suggested to play a role in promoting the interaction between nucleosomes to facilitate chromosome condensation^18^; and H3S86, which was identified in a large-scale proteomics analysis to assess histone PTMs in mouse brain^19^,^20^.

Aside from its identification, no physiological function was attributed to the PTM of H3S86 in the proteomics analysis. Thus, to ascertain the physiological significance of this phosphorylation, we adopted a genetic approach and characterized a mutant fission yeast strain carrying an alanine replacement at S86 (*hht2-S86A*), as well as an alanine replacement at the adjacent S87 (*hht2-S87A*) and double alanine substitutions at both S86 and S87 (*hht2-S86AS87A*). These mutations were introduced into the *hht2*^+^ copy of histone H3 (See Methods below). All three mutants exhibited chromosome missegregation phenotypes. Subsequent analyses with the *hht2-S86AS87A* strain showed that it is viable without salient destabilization of histone H3 proteins, but was accompanied by a disruption to proper chromosome segregation. Interestingly, the *hht2-S86AS87A* mutant negatively impacted the transcription of a GATA-type transcription factor, Ams2. We found that overexpression of Ams2 ameliorated the chromosome segregation defects seen with the *hht2-S86AS87A* mutant. Consistent with a regulatory role of the centromeric locus, the *hht2-S86AS87A* mutant displayed increased histone H4 acetylation at the centromere central core. Overall, our studies provide a role for the uncharacterized serine residues in the histone fold domain of histone H3 in conferring genomic stability by safeguarding the fidelity of chromosome segregation.

## Results

### Mutagenesis of histone H3 serine S86 and S87 results in temperature-sensitive growth defects

Previous crystal structure characterization of the nucleosome reveals that the N-terminal tip of the αII helix within the histone fold domain of histone H3 consists of a pair of conserved juxtaposing serine residues situated at the surface of the nucleosome at positions 86 (S86) and 87 (S87)^2^ (Supplementary Fig. 1a). Using mouse brain tissue, S86 was previously shown to be phosphorylated in histone H3, although the function of this modification was not determined at the time^19^. A threonine-80 (T80) residue located near H3S86 and S87 was recently shown to be phosphorylated and can stack against histone H4 to regulate chromatin compaction during mitosis^18^. Modelling of S86 and S87, however, revealed that these two residues lie in close proximity to the DNA (Supplementary Fig. 1b,c) and showed relatively little overlap with that of nearby alanine-83 (A83) and phenylalanine-100 (F100) of histone H4 (Supplementary Fig. 1d). We therefore set out to elucidate the function of S86 and S87 residues of histone H3 by creating single and double alanine substitutions in a strain bearing only one copy (the most highly expressed) of the three histone H3 genes (*hht2*^+^)^21^,^22^, hereafter referred to as *hht2-S86A*, *hht2-S87A* and *hht2-S86AS87A* (Supplementary Fig. 2a–d). We confirmed by Western analysis that the cellular levels of histone H3 and H4 were not perturbed by this manipulation (Supplementary Fig. 2e).

**Figure 1:**
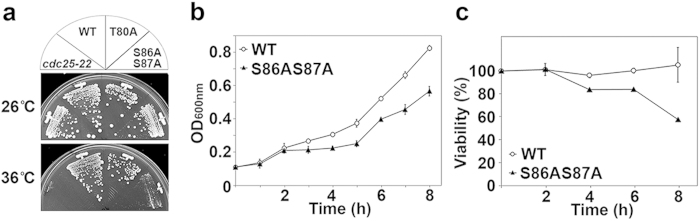
Histone H3 serine-to-alanine double mutant *hht2-S86AS87A* is temperature sensitive. (**a**) Growth of wild-type (WT) cells; cells bearing threonine 80 mutation *hht2-T80A* (*T80A*); cells bearing a double serine 86 and serine 87 mutation (*hht2-S86AS87A*); and the *cdc25-22* strain at 26 °C and 36 °C. (**b**) Growth curve of WT and *hht2-S86AS87A* cells followed over 8 h at 30 °C. (**c**) Cell viability of *hht2-S86AS87A* versus WT cells followed over 8 h after shifting the log-phase cells to 36 °C.

We first examined the effect of the mutations on overall cell growth at different temperatures. We found that the *hht2-S86AS87A* strain showed growth retardation at 36 °C, similar to the temperature-sensitive *cdc25-22* mitotic phosphatase mutant. Another histone mutant, which contained a threonine-to-alanine (T80A) substitution, did not show similar temperature sensitive growth effects, but resembled wild-type (WT) cells at 36 °C (Fig. 1a). The mutant *hht2-S86AS87A* cells showed slower growth rates with increased doubling time (WT = 3 h, *hht2-S86AS87A* = 5 h) even at the permissive temperature (Fig. 1b). When shifted to 36 °C, the viability of *hht2-S86AS87A* cells was reduced as compared with that of WT (Fig. 1c). Similar phenotypic trends were observed in *hht2-S86A* but not *hht2-S87A* (refer below).

We confirmed using immunoblotting that the genetic manipulation did not drastically destabilize the expression of the histone H3, H4 and H2A proteins, with comparable levels observed in the *hht2-S86AS87A* mutant and wild-type (WT) cells (Supplementary Fig. 3a). Using micrococcal nuclease digestion, we showed that the nucleosome ladder pattern of the global chromatin structure was also similar between WT and *hht2-S86AS87A* cells (Supplementary Fig. 3b). We further checked the transcription of several housekeeping genes (*act1*^+^, *pma1*^+^ and *fbp1*^+^) and observed similar levels of transcripts between WT and *hht2-S86AS87A* cells (Supplementary Fig. 4). Finally, we showed that the cell cycle progression was not grossly affected by the mutation in *hht2-S86AS87A* cells (Supplementary Fig. 5a,b). Thus, overall, the *hht2-S86AS87A* mutation did not result in a generally disrupted genomic function.

### *hht2-S86AS87A* mutant exhibits unequal chromosome segregation

**Figure 2:**
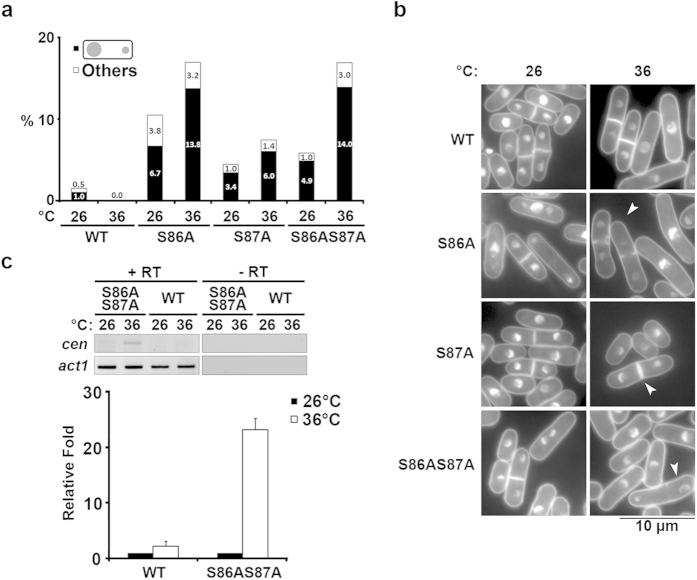
*hht2-S86A*, *hht2-S87A* and *hht2-S86AS87A* mutants exhibit chromosome missegregation defects. (**a**) Frequency of cells exhibiting chromosome missegregation phenotypes in WT, *hht2-S86A, hht2-S87A* and *hht2-S86AS87A* cultures at 26 °C and 36 °C. Black: unequal chromosome segregation; white: all other chromosome missegregation phenotypes. N = 200. Results shown are representative of two experiments. (**b**) Nuclear morphology of strains used in (**a**). Arrowhead depicts a cell with unequal chromosome segregation. Scale bar: 10 μm. (**c**) Top: End-point RT-PCR for centromeric DNA sequence derived-transcript (*cen:* 5′ TTACGCTTCACCTAGTTTCC 3′ and 5′ ATTATTTTCCAGTATGCTGATG 3′) of wild-type (WT) and *hht2-S86AS87A* (S86AS87A) at 26 °C and 36 °C. The result shown is representative of three experiments. The *act1*^+^ (*act1*) gene serves as a loading control. -RT: no reverse transcription control. Bottom: Relative band intensity quantification from three experiments. Error bars represent mean ± S.D.

To investigate the possible cause of the loss in cell viability and altered growth rates, we microscopically observed the *hht2-S86AS87A* mutant cells and noticed that they exhibited a weak temperature-sensitive chromosome missegregation phenotype. At 26 °C (permissive temperature), the cell morphology of the *hht2-S86AS87A* mutant resembled that of WT cells and the proportions of cells within the different cell cycle stages were not significantly different, even though there was a marginal increase (5.9%) in the appearance of binucleated cells with missegregated nuclei in the case of mutant cells (Fig. 2a). This chromosome missegregation phenotype was enhanced (17.0%) at the restrictive temperature (36 °C), such that a higher frequency of defective cells was detected in multinucleated mitotic and post-mitotic mutant cells (Fig. 2a). We noticed that the major class of abnormal cells comprised two nuclei, one large and one small, and this is indicative of unequal partitioning of chromosomes during mitotic segregation (Fig. 2b). Cells exhibiting this unequal chromosome segregation phenotype accounted for the majority of the abnormal cells, with 83% and 82% of total multi-nucleated cells at 26 °C and 36 °C, respectively (4.9% and 14.0%; Fig. 2a).

The *hht2-S86A* mutant also showed a pronounced chromosome missegregation phenotype, approximate to that of the *hht2-S86AS87A* double mutant, but only a slight chromosome segregation defect was noted in the *hht2-S87A* single mutant (Fig. 2a). This observation suggests that, for the most part, *hht2-S86A* contributes to these chromosome missegregation defects, with only a minor defect attributed to the S87 mutation. Due to the possible function implication of both S86 and S87, we performed subsequent analyses mostly using *hht2-S86AS87A* mutant.

The observed chromosome missegregation phenotype is reminiscent of that which arises from defects in the architecture of the inner centromeric core; this is shown in mutants of the centromere-specific histone H3 variant, CENP-A, and the KMN (KNL-1/Mis12 complex/Ndc80 complex) complex component, Mis12^23^,^24^,^25^. Chromatin of the inner centromeric core is normally maintained in a transcriptionally silenced conformation, and its disruption results in transcription derepression^26^. Therefore, we employed reverse transcriptase-polymerase chain reaction (RT-PCR) to assess changes in transcript levels with the variation in temperature (restrictive versus permissive). Total RNA was prepared from WT and *hht2-S86AS87A* cells grown at 26 °C and then shifted to 36 °C for 4 h. Whereas a constant level of actin gene transcript (*act1*) was detected in all cells at both temperatures, the transcript deriving from centromeric locus could only be detected at the restrictive temperature of 36 °C in *hht2-S86AS87A* (23-fold relative to that at 26 °C) (Fig. 2c). Chromatin immunoprecipitation (ChIP) was performed to show that histone H4 lysine 16 (H4K16) acetylation—reported to negatively regulate chromatin compaction^4^—was upregulated at the centromeric region. Consistent with increased transcription at the centromere, we also observed a higher level of histone acetylation at the central centromeric core domain of the mutant cells as compared with WT cells (Supplementary Fig. 6).

Disrupted chromatin integrity at the centromeric core region can occur, for example, as a results of loss-of-function mutations in centromere binding proteins Mis6, Mis17 and Mis18, which in turn increases the susceptibility of the cells towards histone deacetylase inhibitors (HDACi)^27^. Thus, we tested whether the *hht2-S86AS87A* mutant exhibits hypersensitivity towards the HDACi, suberoylanilide hydroxamic acid (SAHA), produced in-house^28^. Interestingly, *hht2-S86AS87A* mutant showed hypersensitivity towards SAHA at permissive temperature (Supplementary Fig. 7a), and exhibited similar unequal chromosome segregation phenotype as that exhibited by the mutant at the restrictive temperature (Fig. 2a,b, Supplementary Fig. 7b,c). These observations lend further support for a mechanistic link between compromised centromeric architecture in the *hht2-S86AS87A* mutant and disrupted chromosome segregation.

### *hht2-S86AS87A* mutant exhibits disrupted Ams2 at both transcriptional and translational levels

Canonical histone H3 is replaced by CENP-A in the nucleosome of chromatin at the inner centromeric core^23^. Previous work has shown that a disruption to the centromeric chromatin results in a reduction in the localization of CENP-A and an increase in the incorporation of histone H3^29^,^30^. In this case, even a slight increase in the cellular level of histone H3 protein is sufficient to destabilize centromeric function and result in a prominent reduction in cell viability^30^. Because of these toxic effects of histone H3 on the integrity of the inner centromeric chromatin, we deemed it unlikely that there would be direct regulation of histone S86 (and/or S87) at the centromeric core. Furthermore, taking clues from the proximity of these two residues to the DNA on the surface of the nucleosome in the 3D structural modelling (Supplementary Fig. 1b,c), we hypothesized that these serine residues may play an indirect role in affecting the transcription and/or translation of genes that encode factors essential for the maintenance of centromeric integrity.

We therefore employed RT-PCR to check the transcript levels of 18 key genes of inner centromeric regulators in the *hht2-S86AS87A* mutant versus WT strains at 26 °C and 36 °C to identify which gene transcripts were affected by the *hht2-S86AS87A* mutation: *ams2*^+^, *cnp1*^+^, *cnp3*^+^, *csm1*^+^, *dad1*^+^, *dad2*^+^, *dad5*^+^, *duo1*^+^, *fta3*^+^, *mis12*^+^, *mis13*^+^, *mis15*^+^, *mis16*^+^, *mis18*^+^, *ndc80*^+^, *nnf1*^+^, *sim3*^+^ and *spc19*^+^^27^,^30^,^31^,^32^,^33^,^34^,^35^,^36^,^37^,^38^. The transcript levels of most of the genes tested were not affected in the *hht2-S86AS87A* strain, with the exception of two genes, *cnp1*^+^and *ams2*^+^ (Supplementary Fig. 8). *cnp1*^+^ encodes centromere protein A (SpCENP-A), which is an important component of the fundamental scaffold of the centromere^24^, whereas Ams2 is a GATA-type transcription factor that also plays a direct role in maintaining SpCENP-A localization at the centromere^30^. These genes appeared to show reduced levels in *hht2-S86AS87A* cells relative to that of WT (Supplementary Fig. 8a,b). However, upon further validation with real-time qRT-PCR, only *ams2*^+^ reproducibly showed a prominent reduction (Supplementary Fig. 8c, *ams2*^+^; 8d, *cnp1*^+^).

**Figure 3:**
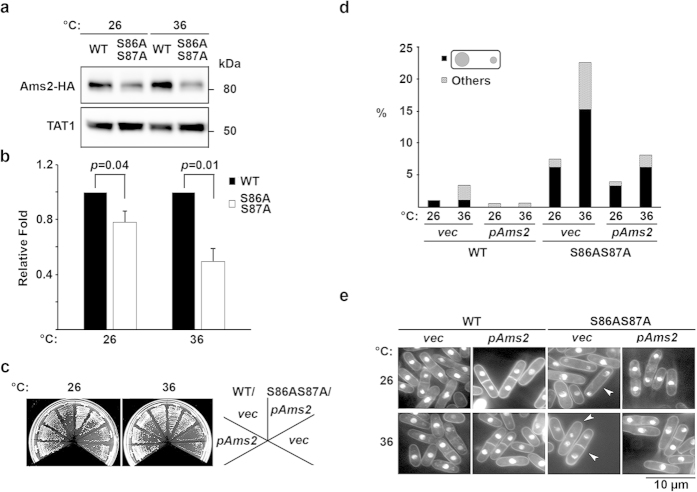
*hht2-S86AS87A* mutation affects Ams2 expression level and results in chromosome segregation defects. (**a**) Protein expression level of 3 × HA-tagged Ams2 (Ams2-HA) in *hht2-S86AS87A* versus WT cells at 26 °C and 36 °C. Result shown is representative of three experiments. (**b**) Mean of relative band intensity quantification from three experiments in (**a**). Black, WT; white, *hht2-S86AS87A*. Error bars represent mean ± S.D. (**c**) Growth of WT and *hht2-S86AS87A* cells transformed with empty vector (*vec*) or those cloned with endogenous promoter-driven *ams2*^+^ gene (*pAms2*) at 26 °C and 36 °C. Growth retardation of *hht2-S86AS87A* was suppressed by the expression of *ams2*^+^ gene. (**d**) Frequency of chromosome missegregation for strains used in (**c**). Black bars, unequal chromosome segregation; hatched bars, cells with other aberrant nuclear morphologies. Results shown are representative of two experiments. N = 200. (**e**) Nuclear morphology of strains used in (**d**). Arrowhead depicts a cell with unequal chromosome segregation. Scale bar: 10 μm.

Next, we asked whether the Ams2 protein was also affected by the *hht2-S86AS87A* mutation. To this end, we constructed a 3 × hemagglutinin (HA)-tagged *ams2*^+^ gene in WT and *hht2-S86AS87A* genetic backgrounds, and employed western blotting to assess Ams2-HA protein levels from cells grown at 26 °C and 36 °C. As expected, the Ams2-HA protein level was reduced in *hht2-S86AS87A*, even though the protein expression was not completely abrogated (Fig. 3a). Interestingly, we noted a 22% reduction in protein levels at 26 °C, which decreased to half that of the WT cells when incubated at 36 °C for 4 h (Fig. 3b). Ams2 has been shown to bind directly to the inner centromeric sequence and *ams2* disruption results in a chromosome missegregation phenotype^30^. Hence, it is possible that the chromosome missegregation phenotype exhibited by the *hht2-S86AS87A* mutant may be a result of the reduction in Ams2 protein levels.

### Suppression of chromosome segregation defects of *hht2-S86AS87A* mutant with ectopic Ams2 expression

To verify the role of Ams2 in the chromosome missegregation phenotype, we ectopically expressed *ams2*^+^ gene using its native promoter and assessed chromosome missegregation in WT and *hht2-S86AS87A* cells. WT cells ectopically expressing *ams2*^+^ (WT, *pAMS2*) grew as well as those containing the empty vector (WT, *vec*) at both 26 °C and 36 °C, showing that the low level of *ams2*^+^ overexpression did not cause any dominant-negative growth retardation (Fig. 3c, left). Interestingly, the ectopic expression of the *ams2*^+^ gene (*pAMS2*) in the *hht2-S86AS87A* strain resulted in a suppression of the growth defect to a degree that almost approximated that of WT cells (Fig. 3c, right), as compared with *hht2-S86AS87A* cells containing the empty vector (*vec*), which showed the expected temperature-dependent growth attenuation and chromosome missegregation phenotype, particularly at 36 °C (7.6% at 26 °C and 22.7% in 36 °C; Fig. 3d). Interestingly, *hht2-S86AS87A* cells expressing *ams2*^+^ showed a 1.9-fold reduction at 26 °C and a 2.8-fold reduction at 36 °C (4% at 26 °C and 8.1% at 36 °C) in chromosome segregation defect, which coincided with the growth suppression defect (Fig. 3d,e). Taken together, these results show that the growth defects and chromosome missegregation phenotype associated with the *hht2-S86AS87A* mutant are, to a large extent, a result of the reduction in Ams2.

### Defective centromeric localization of SpCENP-A^*cnp1-1*^ in *hht2-S86AS87A*

Ams2 functions to maintain centromeric integrity by regulating the precise localization of SpCENP-A (Sp: *Schizosaccharomyces pombe*)^30^. A SpCENP-A mutant (SpCENP-A^*cnp1-1*^), hosting a L87Q mutation in the αII helix of the histone fold domain that formed in the CENP-A-targeting domain (CATD), was shown to delocalize from the centromeric DNA at restrictive temperature readily relocalized upon the ectopic expression of *ams2*^+^^24^,^30^,^39^. Hence, it is possible that the centromeric defects associated with the *hht2-S86AS87A* mutant were due to a mislocalization of CENP-A following Ams2 deregulation (Fig. 3a). We examined this possibility using a FLAG-tagged SpCENP-A expressed from a transformed plasmid (driven by native promoter) in WT and *hht2-S86AS87A* cells in ChIP experiments to assess centromeric binding of SpCENP-A-FLAG but detected no significant change between WT and the *hht2-S86AS87A* mutant at either 26 °C or 36 °C; this shows that the steady state of SpCENP-A localization is not affected in the *hht2-S86AS87A* mutant during G2 (the longest part of fission yeast cell cycle^40^; Supplementary Fig. 9).

**Figure 4:**
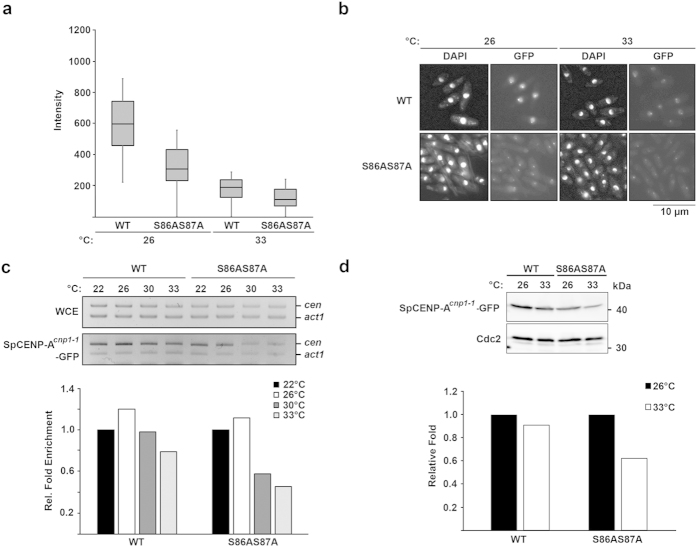
Localization of centromere protein SpCENP-A^*cnp1-1*^-GFP is disrupted in *hht2-S86AS87A*. (**a**) Box plot of SpCENP-A^*cnp1-1*^-GFP intensity measured for WT and *hht2-S86AS87A* cells growing at 26 °C and shifted to 33 °C for 4 h. (**b**) Microscopic observation of SpCENP-A^*cnp1-1*^-GFP and nuclear staining (DAPI) of strains used in (**a**). Scale bar: 10 μm. (**c**) ChIP assay of SpCENP-A^*cnp1-1*^-GFP in WT and *hht2-S86AS87A* cells first grown at 26 °C and then shifted to 22 °C (6 h), 30 °C (4 h), or 33 °C (4 h). ChIP was analysed with competitive PCR using primers specific for the centromere core (*cen:* 5′-TTACGCTTCACCTAGTTTCC-3′ and 5′-ATTATTTTCCAGTATGCTGATG-3′) and control gene (*act1*) (top). The relative fold enrichment shown in the graph below was calculated by comparing the *cen/act1* ratio between ChIP and whole-cell extracts (WCE). The value was normalized according to that at 22 °C for each strain. (**d**) Protein expression of SpCENP-A^*cnp1-1*^-GFP in WT and *hht2-S86AS87A* growing at 26 °C and shifted to 33 °C for 4 h (top). Relative band intensity quantification shown in the bar graph (below). Results shown are the representative of two experiments.

We next investigated whether *hht2-S86AS87A* may synergize with a *cnp1-L87Q* mutation to affect the rate of dissociation of SpCENP-A^*cnp1-1*^protein from the centromere. To this end, we integrated an extra copy of *cnp1-1* that was tagged with a C-terminal GFP under the transcriptional control of the native promoter and integrated at the exogenous *lys1* locus^30^ in both WT and *hht2-S86AS87A* backgrounds. We observed the direct fluorescence associated with the GFP tag and also performed ChIP to assess centromeric binding of the SpCENP-A^*cnp1-1*^protein. Surprisingly, we detected a median reduction in GFP intensity of almost 50% at the permissive temperatures of 26 °C and 33 °C (0.54-fold and 0.66-fold, respectively, relative to WT) (Fig. 4a,b). This microscopic observation was supported by ChIP data, which showed a reduction in SpCENP-A^*cnp1-1*^protein binding at the DNA sequence of the centromeric core (0.45-fold at restrictive (33 °C) relative to permissive temperatures; Fig. 4c), albeit, there was a destabilization of SpCENP-A^*cnp1-1*^protein at 33 °C (0.38 fold reduction relative to 26^o^C) (Fig. 4d). These results indicate that *hht2-S86AS87A* synergizes with the *cnp1-1* mutation, and that the SpCENP-A^*cnp1-1*^protein was preferentially destabilized by the histone H3 mutation.

### Phospho-mimetic S86 mutant exhibits unequal chromosome segregation

**Figure 5:**
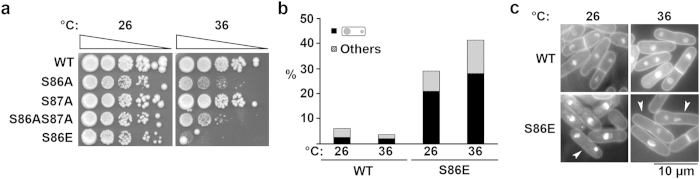
Phospho-mimetic mutant *hht2-S86E* exhibits growth and chromosome segregation defects. (**a**) Growth of WT, *hht2-S86A*, *hht2-S87A*, *hht2-S86AS87A* and *hht2-S86E* at 26 °C and 36 °C. Cells were 10-fold serially diluted and spotted. Triangle indicates the direction of spotting from most (left) to least (right)-concentrated cultures. (**b**) Frequency of cells exhibiting a chromosome missegregation phenotype in WT and *hht2-S86E* cultures at 26 °C and 36 °C. Black bars, unequal chromosome segregation; hatched bars, cells with other aberrant nuclear morphologies. Results shown are representative of two experiments. N = 200. (**c**) Nuclear morphology of strains used in (**b**). Arrowhead depicts cells with unequal chromosome segregation. Scale bar: 10 μm.

Finally, we interrogated the phenotype of a constitutive, phospho-mimetic of S86. We constructed a strain following similar procedures described above to substitute serine with glutamic acid (S86E) in the genomic copy of *hht2*^+^ (Supplementary Fig. 2). Unexpectedly, we observed that the *hht2-S86E* mutant also exhibited a prominent temperature-sensitive growth retardation, which was more severe than the loss-of-function with the double alanine mutant (Fig. 5a). The *hht2-S86E* mutant also exhibited a chromosome missegregation phenotype both at 26 °C and 36 °C, although more salient in the latter (Fig. 5b), with unequal segregation of 21% noted at 26 °C and 28.1% at 36 °C (Fig. 5b,c). These results indicate that the maintenance of a constitutive negative charge at this serine residue, which mimics the constitutive phosphorylation of the serine residue, is also toxic to the cells, similar to the loss of function of the serine residue.

## Discussion

Histone proteins are subjected to extensive PTMs, with phosphorylation events important for recruiting specific targets that are essential for normal cell processes, including the maintenance of genomic integrity. Histone H3 was recently shown to have a phosphorylation event at S86 of the histone fold domain^19^,^20^; yet, the relevance of this modification is unknown. In this work, we investigated the role of two adjacent serine residues—S86 and S87—using loss-of-function, site-directed mutagenesis, and show that the loss of H3S86 and H3S87 function results in unequal chromosome segregation and compromised transcriptional silencing of the chromatin at the centromeric core. This loss of function also results in a decrease in the level of the GATA-type transcription factor Ams2, a protein that safeguards chromatin integrity of the inner core of centromere. We show that the overexpression of Ams2 could partially restore the normal phenotype of the mutant cells. Furthermore, we detected a reduction in the binding of the specialized histone H3 variant CENP-A at the central centromeric core. Our findings thus reveal a surprising mechanism whereby fission yeast histone H3—whose upregulated presence in the centromeric core will disrupt CENP-A-chromatin—is required for safeguarding CENP-A-chromatin via the transcriptional regulation of Ams2, which acts to regulate CENP-A deposition^30^.

Although S86 and S87 reside adjacent, it is interesting to observe such functional dichotomy with regard to their impact on precise chromosome segregation. Whereas *hht2-S86A* exhibited a temperature-sensitive growth retardation and prominent chromosome segregation defect, *hht2-S87A* showed a WT-like growth and only minor chromosome missegregation. As observed from the 3D model, S86 appeared to be closer to the DNA helix than S87, and thus it is possible that, because of its location, S86 may modulate the interaction of DNA with the nucleosomal surface. If S86 were phosphorylated, then the addition of the negatively charged phospho-group may result in the opening up of the chromatin in response to electrostatic repulsion with the negatively charged DNA. This change may in turn facilitate localized accessibility of the underlying DNA sequence by RNA polymerase II. Conversely, the site-directed disruption of S86 would prohibit the release of DNA from the nucleosomal surface and restrict DNA accessibility and, consequently, transcription (Supplementary Fig. 10). Future experiments will be needed to show the presence of S86ph on the fission yeast chromatin, particularly at the promoter of the *ams2*^+^ gene.

A reduction in the transcription level of the *ams2*^+^ gene was not temperature dependent, as a similar level of transcript was detected both at 26 °C and 36 °C; from these results we infer that transcriptional deregulation is insufficient to explain the temperature-sensitive defect in the *hht2-S86AS87A* mutant. Conversely, a reduction in the level of the Ams2 protein was temperature dependent. Ams2 is reported to be post-translationally phosphorylated, ubiquitinated and targeted for proteasomal degradation^41^. A reduction in Ams2 protein levels at the restrictive temperature may be a consequence of its unrestrained and excessive degradation. Alternatively, reduced Ams2 levels may be due to a reduction in the translation of the *ams2*^+^ transcript. Future experiments, which include examining the level of Ams2 protein in the presence of proteasome or ribosome inhibitors, would shed more light on the likelihood of these potential mechanism(s).

The *hht2-S86AS87A* mutation did not affect the centromeric association of WT SpCENP-A with DNA, but greatly destabilized the SpCENP-A^*cnp1-1*^ protein, which harbors a L87Q^24^,^30^ mutation in the histone fold domain. This mutation lies in the CENP-A-targeting domain, previously identified in budding yeast and human CENP-A proteins^42^. This observation suggests that the mechanism regulated by H3S86 and H3S87 is probably not a major pathway like the canonical mechanisms that involve centromeric proteins Mis6, Mis16, Mis18 and Scm3^32^,^43^,^44^. However, S86 (and S87) appears to play a synergistic role in determining SpCENP-A localization, which is only apparent when SpCENP-A is destabilized through the incorporation of this mutation.

The reduction in SpCENP-A^*cnp1-1*^ protein in *hht2-S86AS87A* cells may contribute to the lower centromeric binding observed in this mutant. However, it is also possible that a lower protein stability may be caused by degradation of the delocalized SpCENP-A^*cnp1-1*^ protein, in a manner reminiscent of the degradation of overexpressed SpCENP-A, which prevents mistargeting, as shown recently^45^. If the latter is true, then the decreased level of the SpCENP-A^*cnp1-1*^ protein will be an outcome that follows the centromeric delocalization of the protein. These two possibilities will be clarified in future studies.

In conclusion, our characterization of the *hht2-S86AS87A* mutant has uncovered a mechanism whereby transcription of a centromere-acting factor controlling chromosome segregation fidelity is modulated through S86 in histone H3. Our work thus showed an unexpected positive regulation imposed by histone H3 on the integrity of the centromeric core domain and underscores a mechanism in which histone H3 can serve a positive role in stabilizing the chromatin foundation of the fission yeast centromeric core domain.

## Methods

### Strains and media

Standard manipulation of fission yeast cells was followed^46^. Yeast strains used in this study are listed in Supplementary Table 1. The strains were constructed based on standard PCR-based procedures described in Supplementary Fig. 2a–d. The exact sequences of primers used are listed in Supplementary Table 2. Fission yeast cells were cultured using complete YEA media (3% glucose, 0.5% yeast extract, 75 mg/L adenine) and minimal EMM2. Tetrad dissection was carried out with an MSM micromanipulator (Singer Instrument, Watchet, Somerset, UK) to generate strains with different genetic backgrounds.

### Viability and growth rate assay

Cultures were incubated at 36 °C for 2-h intervals over a total of 8 h. From these cultures, 500 cells were plated on YEA plates and cell viability was assessed by counting colonies formed after incubation for 4–5 days at 26 °C. Cell growth rate was determined by measuring the OD (optical density) at 600 nm every hour for cells growing in rich YEA medium at 30 °C.

### Spot assays

Asynchronous mid-log–phase growing cells were 10-fold serially diluted, and 3 μl of cultures were spotted on YEA plates incorporated with 10 mM of SAHA, which was synthesized in-house and verified by liquid chromatography and ^1^H and ^13^C nuclear magnetic resonance^28^. Plates were incubated at 26 °C for 7 days before pictures were taken. For identification of the effect of the phospho-mimetic S86 (S86E), diluted cells were spotted on YEA plates and incubated at 26 °C and 36 °C for at least 3 days.

### Antibodies

Primary antibodies against α-HA (12CA5; Roche Applied Science, Basel, Switzerland), α-Cdc2 (sc-53; Santa Cruz Biotechnology, Dallas, TX), α-H3 (06-755; Millipore, Billerica, MA), α-H4 (05-858; Millipore), α-H2A (07-146; Millipore), α-H4K16ac (07-329; Millipore), α-FLAG (018-22381; Wako Pure Chemical Industries, Osaka, Japan) and α-GFP (11814460001; Roche Applied Science) were obtained from commercial sources. α-TAT1 was previously reported^47^.

### Immunoblotting

Asynchronous cells in log phase (OD_600nm_, 0.5) were collected by centrifugation, and total proteins were prepared using trichloroacetic acid (TCA; Sigma-Aldrich, St. Louis, MO) precipitation, as described^48^. Protein precipitates were resuspended using Laemmli buffer (0.1 M Tris-HCl, pH6.8, 4% SDS, 20% glycerol, 0.1 M DTT), and heated at 100 °C for 4 min. Proteins were separated using polyacrylamide gels and transferred to Hybond ECL nitrocellulose membranes (GE Healthcare, Little Chalfont, UK). Primary antibodies were used at room temperature for 1 h followed by incubation with goat-anti-mouse IgG-HRP (sc-2005; Santa Cruz Biotechnology) or goat-anti-rabbit IgG-HRP (sc-2004; Santa Cruz Biotechnology) at room temperature for 1 h, followed by chemiluminescence assay using Amersham ECL Prime and ImageQuant LAS 4000 imager (GE Healthcare).

### Chromatin Immunoprecipitation (ChIP)

ChIP was performed as previously described for the inner centromeric sequence *cen* with *act1* as control^30^ (Supplementary Table 3). Bands from the competitive PCR were quantified using ImageQuant TL software.

### Quantification of Ams2 mRNA and protein level

Quantitative PCR (qPCR) was performed using StepOne Real-Time system (Life Technologies, Carlsbad, CA), SYBR Green PCR Master Mix (Applied Biosystems, Life Technologies, Foster City, CA) and the appropriate primers to quantify the expression level of Ams2 mRNA. Data are the mean of three experiments normalized to actin (*act1*), which was used as an internal control. Quantification of the 3×HA-tagged Ams2 protein was measured using immunoblotting and band intensity was measured using ImageJ software^49^.

### RT-PCR of centromere-related genes

Total RNA was isolated using Trizol reagent (Life Technologies), as previously described^40^, and followed by DNase I digestion steps (M0303; New England Biolabs, Ipswich, MA). First-strand cDNA synthesis was done using primers that were complementary to the sense transcript and the One-Step RT-PCR kit (Qiagen, Venlo, Netherlands). A second primer was added for subsequent PCR amplification steps. Triplicate experiments were performed. Primer sequences are listed in Supplementary Table 3.

### Micrococcal nuclease (MNase) digestion

Asynchronous mid-log–phase growing cells were growing at 26 °C before shifting to 36 °C for 4 h. The MNase assay was performed on collected cells as previously described^50^, with slight modifications. 3.8 Units of MNase (M0247S, New England Biolabs, Ipswich, MA) was added to each samples and treated for 0, 3, 6 and 9 min. Purified DNA was separated in agarose gels and stained with ethidium bromide (Life Technologies).

### Microscopic observation

Cellular phenotype was observed using 50 μg/ml 4′,6-diamidino-2-phenylindole (DAPI) (Life Technologies), as previously described^51^. Cells were fixed with 10% glutaraldehyde (Sigma-Aldrich), washed, and resuspended with phosphate-buffered saline (PBS). For observation of SpCENP-A^*cnp1-1*^-GFP, methanol fixation was done by harvesting cells using glass filter (GF/C, GE Healthcare) and kept in methanol (Sigma-Aldrich) for 2 h at −80 °C. Cells were rehydrated using PBS and DAPI was added before observed under microscope. A Nikon Eclipse Ti-E fluorescence microscope (Nikon, Tokyo, Japan) was employed. Intensity of GFP foci was determined using NIS-Element software (Nikon, Tokyo, Japan) (Supplementary Fig. 11).

### 3D structures modelling of nucleosome

Accelrys Discovery Studio 3.5 Visualizer (San Diego, CA) was used to examine the three-dimensional structure of the nucleosome core particle (Protein Data Bank entry 1AOI).

## Additional Information

**How to cite this article**: Lim, K.K. *et al.* Mutation of histone H3 serine 86 disrupts GATA factor Ams2 expression and precise chromosome segregation in fission yeast. *Sci. Rep.* **5**, 14064; doi: 10.1038/srep14064 (2015).

## Supplementary Material

Supplementary Information
