# Supplementary material for: Mutation of histone H3 serine 86 disrupts GATA factor Ams2 expression and precise chromosome segregation in fission yeast
Source: Sci Rep. 2015 Sep 15;5:14064. doi: 10.1038/srep14064 (PMC4570208; doi:10.1038/srep14064)
Supplement: Supplementary Information [file srep14064-s1.pdf]

Supplementary Information
(11 Figures and 3 Tables)

Mutation of histone H3 serine 86 disrupts GATA factor Ams2 expression and precise chromosome segregation in fission yeast

Kim Kiat Lim[1,2], Terenze Yao Rui Ong[1,2], Yue Rong Tan[1,2], Eugene Guorong Yang[5], Bingbing Ren[1,2], Kwi Shan Seah[1,2], Zhe Yang[1,2], Tsu Soo Tan[6], Brian W. Dymock[5] and Ee Sin Chen[1,2,3,4,]*

[1]Department of Biochemistry, School of Medicine, [2]National University Health System (NUHS), [3]Synthetic Biology Research Consortium, [4]NUS Graduate School for Integrative Sciences and Engineering, [5]Department of Pharmacy, Faculty of Science, National University of Singapore, Singapore. [6]School of Chemical & Life Sciences, Nanyang Polytechnic, Singapore.

*To whom correspondence should be addressed. Tel: +65-6516-5616; Email: bchces@nus.edu.sg

Present address: [T.Y.R. Ong] DNA Profiling Laboratory @ Synapse, Health Sciences Authority, Ministry of Health, Singapore 138623, Singapore

Key words: Fission yeast, chromosome segregation, histone H3, Ams2, Centromere

Supplementary Figures and Table Legends

Supplementary Figure 1 Histone H3 serine 86 and serine 87 are conserved residues located in the vicinity of DNA within the nucleosome. (a) Alignment of the histone H3 protein sequences between human (*H. sapiens*), fission yeast (*S. pombe*), budding yeast (*S. cerevisiae*), nematode (*C. elegans*), fruit fly (*D. melanogaster*) and plant (*A. thaliana*), with a schematic representation of the secondary structure of histone H3, as previously described[2]. Position of histone H3 serines 86 (S86) and 87 (S87) are denoted by asterisks. (b) Three-dimensional structural models of the nucleosome particle showing only a histone H3 (blue) /H4 (green) pair with respect to the DNA helix (white). Position of S86 and S87, and threonine 80 (T80) are highlighted. (c) Magnification of (b). (d) Solvent surfaces of histone H3 S86 and S87 do not overlap with that of nearby alanine 83 (A83) and phenylalanine 100 (F100) of histone H4.

Supplementary Figure 2 Construction of Histone H3 point mutation strains. (a) DNA fragment containing the desired mutation at *hht2* (*) was obtained by PCR and subsequently transformed into strain with genotype Δ*hht1::kanMX6* Δ*hht2::ura4*$^{+}$. (b) Successful transformants were obtained via homologous recombination replacement of the *ura4*$^{+}$ at the *hht2* locus and selected by growth on media plates containing 5-FOA. (c) The strain obtained from (b) was then crossed with Δ*hht2::ura4*$^{+}$ Δ*hht3::kanMX6*. (d) Progeny cells with the desired genotype of Δ*hht1::kanMX6 hht2-** Δ*hht3::kanMX6* obtained from the cross were first checked by PCR with primers that can differentiate between wild-type (WT) and the disrupted gene. The presence of the mutation was further confirmed by DNA sequencing. (e) Histone H3 and H4 levels of *972* h^{-} WT and the WT (WT: Δ*hht1::kanMX6* Δ*hht3::kanMX6*) used in current study. Cdc2 detected using α-PSTAIRE was employed as a loading control.

Supplementary Figure 3 Alanine site-directed mutagenesis in histone H3 serine 86 and serine 87 does not induce global disruption to histone and chromatin structure. (a) Protein levels of histone H3 (top), H4 (middle), and H2A (bottom) and the corresponding Cdc2 loading control (detected using anti-PSTAIRE antibody) in wild-type (WT), *hht2-T80A* and *hht2-S86AS87A* mutant cells grown at 26°C and 36°C. (b) Micrococcal nuclease digestion patterns in total chromatin from WT and *hht2-S86AS87A* cells at 26°C and 36°C. Results shown are the representative of two experiments.

Supplementary Figure 4 Transcription level of three house-keeping genes—*act1*$^+$, *pma1*$^+$ and *fbp1*$^+$—were not affected by the *hht2-S86AS87A* mutation relative to that of WT. The transcription level of the three-house keeping genes were assessed by end-point RT-PCR either at 26°C or 36°C. –RT: no reverse transcription control. Results shown are the representative of two experiments.

Supplementary Figure 5 Cell profiling at various stages of the cell cycle in WT and *hht2-S86AS87A* cultures. (a) Frequency of cells with a single nucleus (white, G2-phase) or two nuclei (black) in WT and *hht2-S86AS87A*. (b) Frequency of double-nucleated cells in (a) without a septum (White, M-early S-phase) or with a septum (Black, mid-late S-phase). Error bars represent mean ± S.D. of three experiments. Statistical significance was determined using a Student's t-test. N=200; $p<0.05$ is significant.

Supplementary Figure 6 Increase H4K16 acetylation observed at the centromere of *hht2-S86AS87A*. Chromatin immunoprecipitation of histone H4 acetylated at lysine 16 (H4K16ac) in *hht2-S86AS87A* and WT at an inner centromeric sequence (*cen*) relative to *act1*$^+$ (*act1*) control, at 26°C and 36°C. WCE, whole-cell extracts. Results shown are the representative of two experiments.

Supplementary Figure 7 Deregulation of acetylation level affects chromosome segregation in *hht2-S86AS87A*. (a) Wild-type (WT) and *hht2-S86AS87A* cells were 10-fold serially diluted and spotted onto plates containing 0 or 10 mM suberoylanilide hydroxamic acid (SAHA). (b) Frequency of chromosome missegregation in *hht2-S86AS87A* and WT in the presence or absence of 10 mM SAHA. Black bars, unequal chromosome segregation; hatched bars, cells with other aberrant nuclear morphologies. N=150. Results shown are representative of two experiments. (c) Cell phenotype of (b). Arrowhead depicts cells with unequal chromosome segregation. Scale bar: 10 μm.

Supplementary Figure 8 Transcription of genes encoding factors that maintain inner centromeric chromatin integrity in the histone H3 serine–alanine double mutant (*hht2-S86AS87A*). (a) End-point RT-PCR was employed to screen for genes transcriptionally affected by the *hht2-S86AS87A* mutation. –RT, no reverse transcription control; WT, wild-type. Shown is the representative of two experiments. (b) Quantification of band intensity in (a). (c) Real time qRT-PCR verification of the transcription of *ams2*$^+$ (*: p<0.05), and (d) *cnp1*$^+$ gene. Error bars represent mean ± S.D. of three experiments.

Supplementary Figure 9 Localization of SpCENP-A^{Cnp1}-FLAG to centromere core is not affect in *hht2-S86AS87A*. Chromatin immunoprecipitation (ChIP) of SpCENP-A^{Cnp1}-FLAG in *hht2-S86AS87A* and wild-type (WT) at an inner centromeric sequence (*cen*) relative to *act1*$^+$ (*act1*) control, at 26°C and 36°C. WCE, whole cell extracts. (b) Relative fold enrichment at 36°C as compared with that at 26°C in WT and *hht2-S86AS87A*. Results shown are the representative of two experiments.

Supplementary Figure 10 Schematic representation of how histone H3S86 regulates centromeric integrity. Serine 86 (S86) may be phosphorylated at the promoter and at the start of

the Ams2 open-reading frame (ORF) to regulate the accessibility of the transcribing RNA polymerase (RNA Polymerase II). The resultant Ams2 protein maintains the inner centromeric chromatin by modulating the loading of the centromere protein, CENP-A, which is essential for centromere stability and normal chromosomal segregation. In the case of the alanine-substituted mutant, S86A, the inner centromeric chromatin becomes destabilized, resulting in an opened conformation characterized by high acetylation and an upregulation of the centromeric DNA sequence derived-transcript; this, in turn, leads to aneuploidy (Mis) in the S86A mutant.

Supplementary Figure 11 Methods used to determine SpCENP-A$^{cnp1\text{-}1}$-GFP foci intensity. (a) A measuring line (red) was drawn across the SpCENP-A$^{cnp1\text{-}1}$-GFP foci (bright spot). (b) We used the Nikon NIS-Element software to generate an intensity graph, with the peak correspond to the bright GFP foci. (c) The intensity of the foci was determined by measuring the height from the top of the peak to an arbitrarily determined cut-off point at 200. The same measuring profile was used for different samples to maintain consistency.

Supplementary Table 1 *Schizosaccharomyces pombe* strains used in this study

Supplementary Table 2 Primers employed for site-directed mutagenesis

Supplementary Table 3 Primers used for RT-PCR and ChIP analysis

Supplementary Table 1. *Schizosaccharomyces pombe* strains used in this study

Strain	Genotype	Figure
972	*h-*	S2
SPKK99	*h⁻ Δhht1::kanMX6 Δhht3::kanMX6 ura4-D18 leu1-32 ade6-M210*	1, 2,, 5, S2, S3, S4, S5, S7, S8
SPOT48	*h⁻ hht2-S86AS87A Δhht1::kanMX6 Δhht3::kanMX6 ura4-D18 leu1-32 ade6-M210 his2*	1, 2, 5a, S3, S4, S5, S7, S8
SPOT30	*h⁻ hht2-T80A Δhht1::kanMX6 Δhht3::kanMX6 ura4-D18 leu1-32 ade6-M210 his2*	1a, S3a
SPKK1663	*h⁻ hht2-S86A Δhht1::kanMX6 Δhht3::kanMX6 ura4-D18 leu1-32 ade6-M210 his2*	2, 5a
SPKK1665	*h⁺ hht2-S87A Δhht1::kanMX6 Δhht3::kanMX6 ura4-D18 leu1-32 ade6-M210 his2*	2, 5a
SPKK1696	*h⁻ hht2-S86E Δhht1::kanMX6 Δhht3::kanMX6 ura4-D18 leu1-32 ade6-M210 his2*	5
SPKK914	*h⁺ ams2*-3×HA-*kanMX6 Δhht1::kanMX6 Δhht3::kanMX6 ura4-D18 leu1-32 ade6-M210 his2*	3a, b
SPKK1417	*h⁻ ams2*-3×HA-*kanMX6 hht2-S86AS87A Δhht1::kanMX6 Δhht3::kanMX6 ura4-D18 leu1-32 ade6-M210*	3a, b
cdc25-22	*h⁺ cdc25-22 ura4-D18 leu1-32 ade6-M210*	1
SPKK1442	SPKK99, pSK248 plasmid	3c-e
SPKK1443	SPKK99, pSK248 plasmid	3c
SPKK1444	SPKK99, Native promoter-Ams2 plasmid	3c-e
SPKK1445	SPKK99, Native promoter-Ams2 plasmid	3c
SPKK1450	SPOT48, pSK248 plasmid	3c-e
SPKK1451	SPOT48, pSK248 plasmid	3c
SPKK1452	SPOT48, Native promoter-Ams2 plasmid	3c-e
SPKK1453	SPOT48, Native promoter-Ams2 plasmid	3c
SPKK1411	SPOT99, Native promoter-Cnp1-3×FLAG plasmid	S6, S9
SPKK1416	SPOT48, Native promoter-Cnp1-3×FLAG plasmid	S6, S9
SPKK1717	*h⁻ Δhht1::kanMX6 Δhht3::kanMX6 cnp1-1-GFP*[*lys1⁺*] *ura4-D18 leu1-32 ade6-M210 his2*	4
SPKK1718	*h⁻ hht2-S86AS87A Δhht1::kanMX6 Δhht3::kanMX6 cnp1-1-GFP*[*lys1⁺*] *ura4-D18 leu1-32 ade6-M210 his2*	4

Supplementary Table 2. Primers employed for site-directed mutagenesis

Mutation	Sequence (forward / reverse)
T80A	G GAT TGG AAA CGC AAG tca GCC TTA AAA TCT TGG GCA AT
	AT TGC CCA AGA TTT TAA GGC tga CTT GCG TTT CCA ATC C
S86AS87A	AAG AGC ACC AAT TGC ggc ggc TTG GAA ACG CAA GTC AGT
	ACT GAC TTG CGT TTC CAA gcc gcc GCA ATT GGT GCT CTT
S86A	ACT GAC TTG CGT TTC CAA gcc TCC GCA ATT GGT GCT CTT
	AAG AGC ACC AAT TGC GGA ggc TTG GAA ACG CAA GTC AGT
S87A	GAC TTG CGT TTC CAA TCC gcc GCA ATT GGT GCT CTT CAA
	TTG AAG AGC ACC AAT TGC ggc GGA TTG GAA ACG CAA GTC
S86E	ACT GAC TTG CGT TTC CAA gaa TCC GCA ATT GGT GCT CTT
	AAG AGC ACC AAT TGC GGA ttc TTG GAA ACG CAA GTC AGT

Supplementary Table 3. Primers used for RT-PCR and ChIP analysis

Gene	Primer ID	Sequence (forward / reverse)
act1	Tact1.Fw	GAGTCCAAGACGATACCAGTG
	Tact1.Rv	GGCATCACACTTTCTACAACG
ams2	Ams2_O1858.Fw	TCCAAAACCAACTACCTTAC
	Ams2_O2137.Rv	ACTTGGCATCACCAAATCTG
cnp1	Cnp1F	TTGTACGAGAGATATCTTCAG
	CNN	TGAATTCTCGAGCGGCCGCGAGCACCACGAATCCTC
cnp3	Cnp3_O1822.Fw	CAAGGAGGAGCCATCTTTTG
	Cnp3_OE.Rv	ACACTATTAGGATCCTCATCGTTCGTTTGGAAAAT
csm1	Csm1_O598.Fw	ACTTATAAAGGAGCTTTTCGG
	Csm1_OE.Rv	CCATAGTTAGGATCCTTAATCTTGCAAAGCTTTCATT AAT
dad1	Dad1_NdeI.Fw	GGAATTCCATATGGATATTACTGAAAACATAC
	Dad1_BamHI.Rv	CGGGATCCTCAAGGTGCATCTAGCATTTC
dad2	dad2F	ACAAATAGAAACGCTTGCTGC
	Dad2_OE.Rv	AGAGTATTTGGATCCTTATACCTCTTCAACATCGCCT
dad5	dad5F	TGCGTCGTTCTACCATTGTG
	dad5NR	TGAAGAGCACCAAGACGTCG
duo1	Duo1F	AGCCGAAGAGCAGAGAAAG
	Duo1_BamHI.Rv	CGGGATCCTAACCCGAGAACGCACAT
fbp1	Fbp1_O608.Fw	CTGATATTGGCGAATTCATC
	Fbp1_O965.Rv	GGTACCAAATCCAGAATGC
fta3	Fta3_O354.Fw	CGAAAGCAGTATTACCTTCAC
	Fta3_OE.Rv	ACAAAATAAGGATCCTTAATAGGAACTATAATTGTCT TCACG
mis12	Mis12_orf_fw	GATCAACATTACGAACCAACTG
	Mis12_O343.Rv	CTTGAATTGCTTGATCCATG
mis13	Mis13_O694.Fw	CTTGATAAACAGGACTCTCC
	Mis13_OE.Rv	CAGATTAATGGATCCTTAAACTGATTCGTTTGAGGAA G
mis15	Mis15_O1026.Fw	CACACATTCTCAAGTGCTTG
	Mis15_OE.Rv	ATATTTAGCGGATCCTTAGTTAATCTCTTGTTGTCCGA G
mis16	Mis16_O1105.Fw	TCAACGGTTGCATACCCTG
	Mis16_OE.Rv	TTTCTATGGGGATCCTTACTCCAGATCCCTAGGAGAA
mis18	Mis18_O523.Fw	CCAACTAGGTAATAAGACCG
	Mis18_OE.Rv	TTTCTAGCGGGATCCTTATTTCCTCGTTCGTTTTG
ndc80	Ndc80_O1605.Fw	CTAAGAGGTATAGAATCTCG
	Ndc80_OE.Rv	TACGATGAAGGATCCTTACAGTTCCGAACGAGATAG G
nnf1	Nnf1_O266.Fw	ATGGAGAGTCAGAGGTTGAC
	Nnf1_OE.Rv	AGAAATTTAGGATCCTCAACGTTCTATATTTCCTTGTT C

pma1	Pma1_O2114.Fw	GTCTCTGGCTTATCATTCG
	Pma1_O2433.Rv	ACGAGTAACGAAAATCAACC
sim3	Sim3_O908.Fw	CGTTAACTAGCGATCTTGAG
	Sim3_OE_Not1.Rv	AAGGAAAAAAGCGGCCGCATCCTTCTTTTTCTTATCT TTAGG
spc19	Spc19F	GGCATTTCAACGATTGGAAG
	Spc19_BamHI.Rv	CGGGATCCTTAATGACCTCGTTTTTGTTG

Supplementary Figure 1

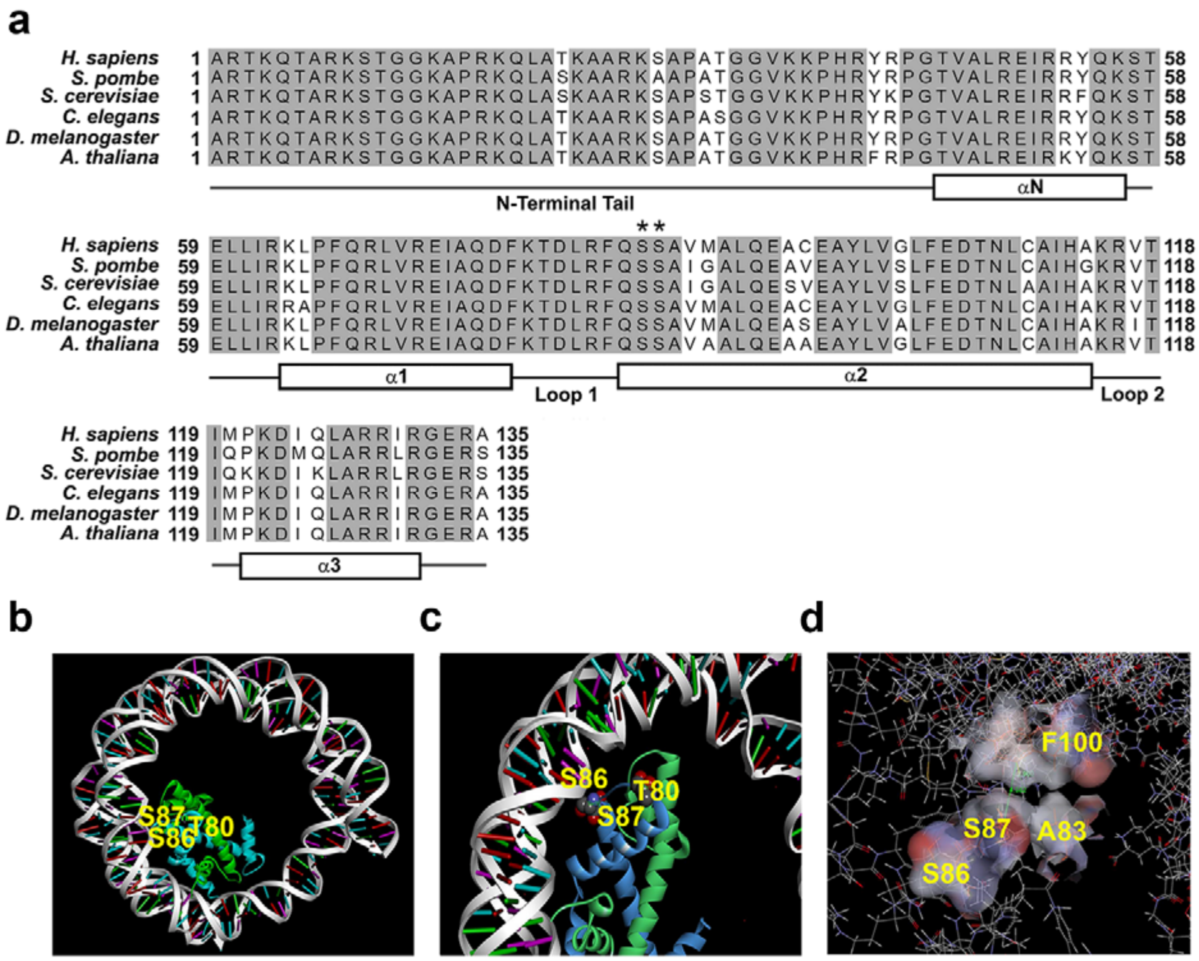

Supplementary Figure 2

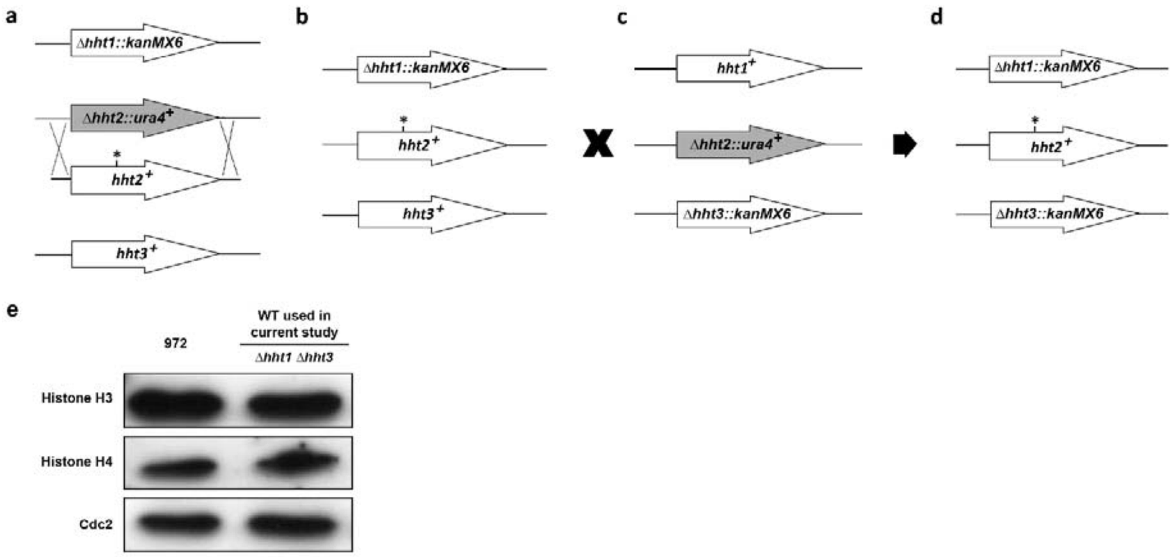

Supplementary Figure 3

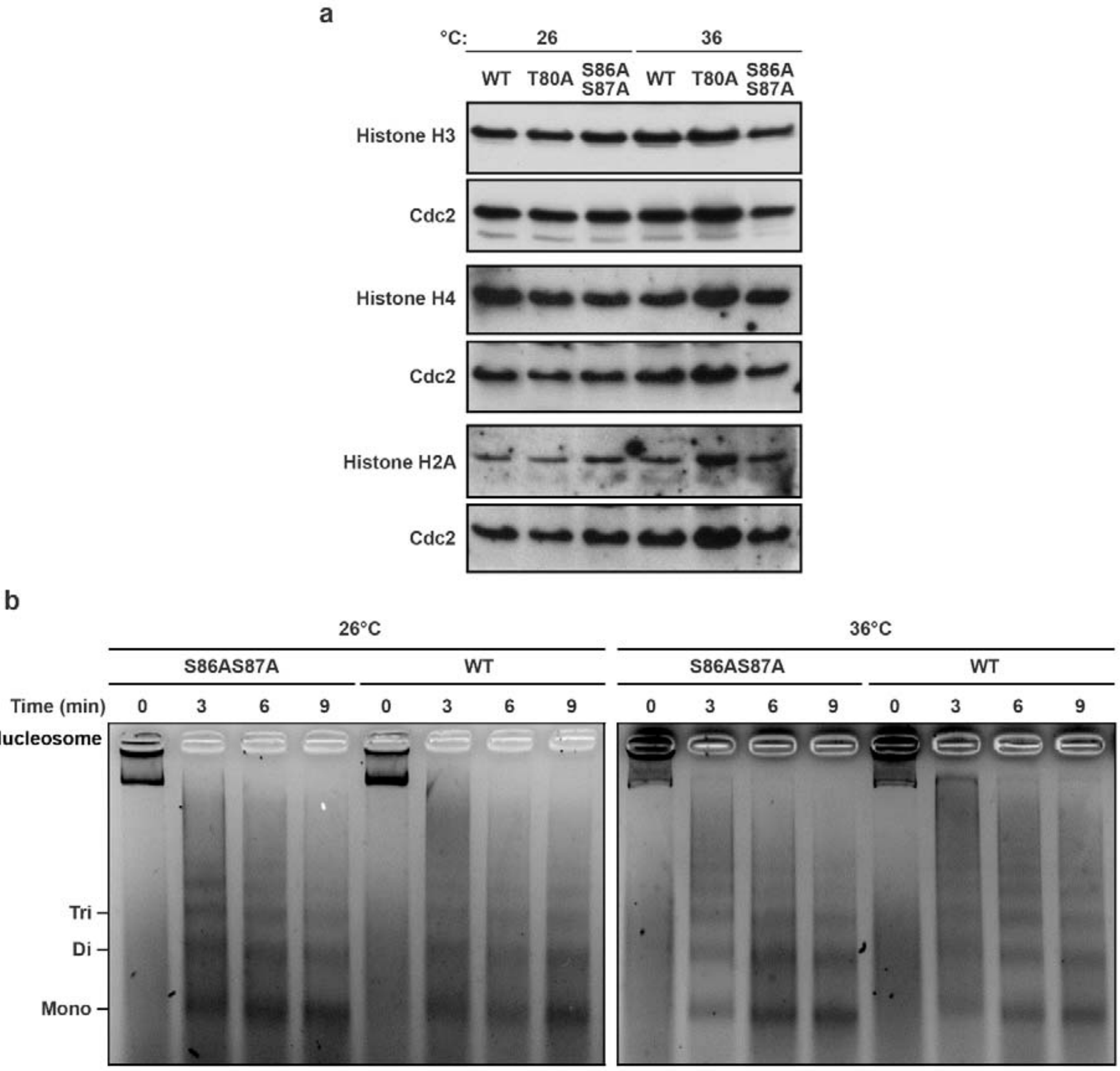

Supplementary Figure 4

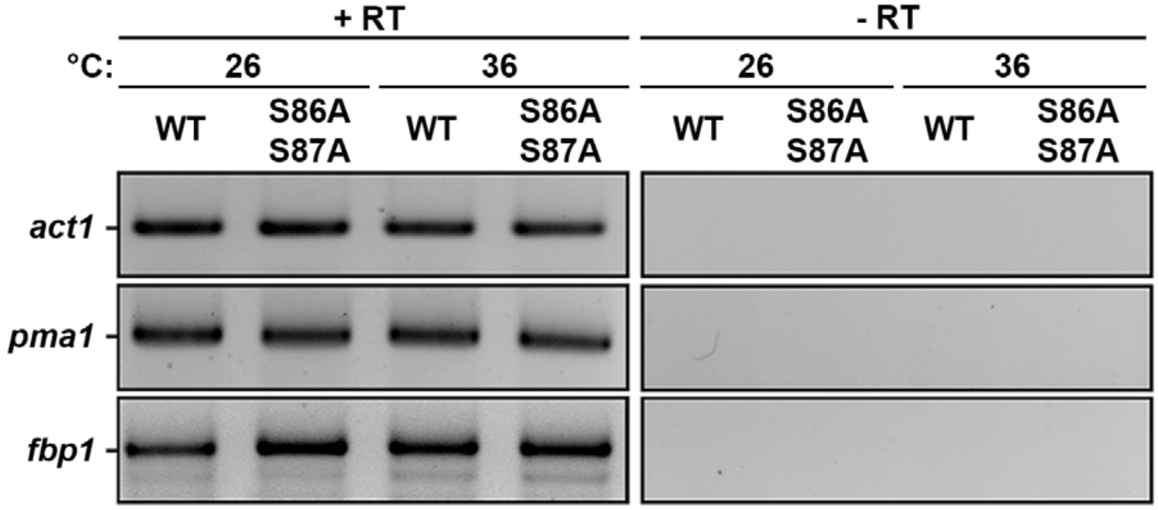

Supplementary Figure 5

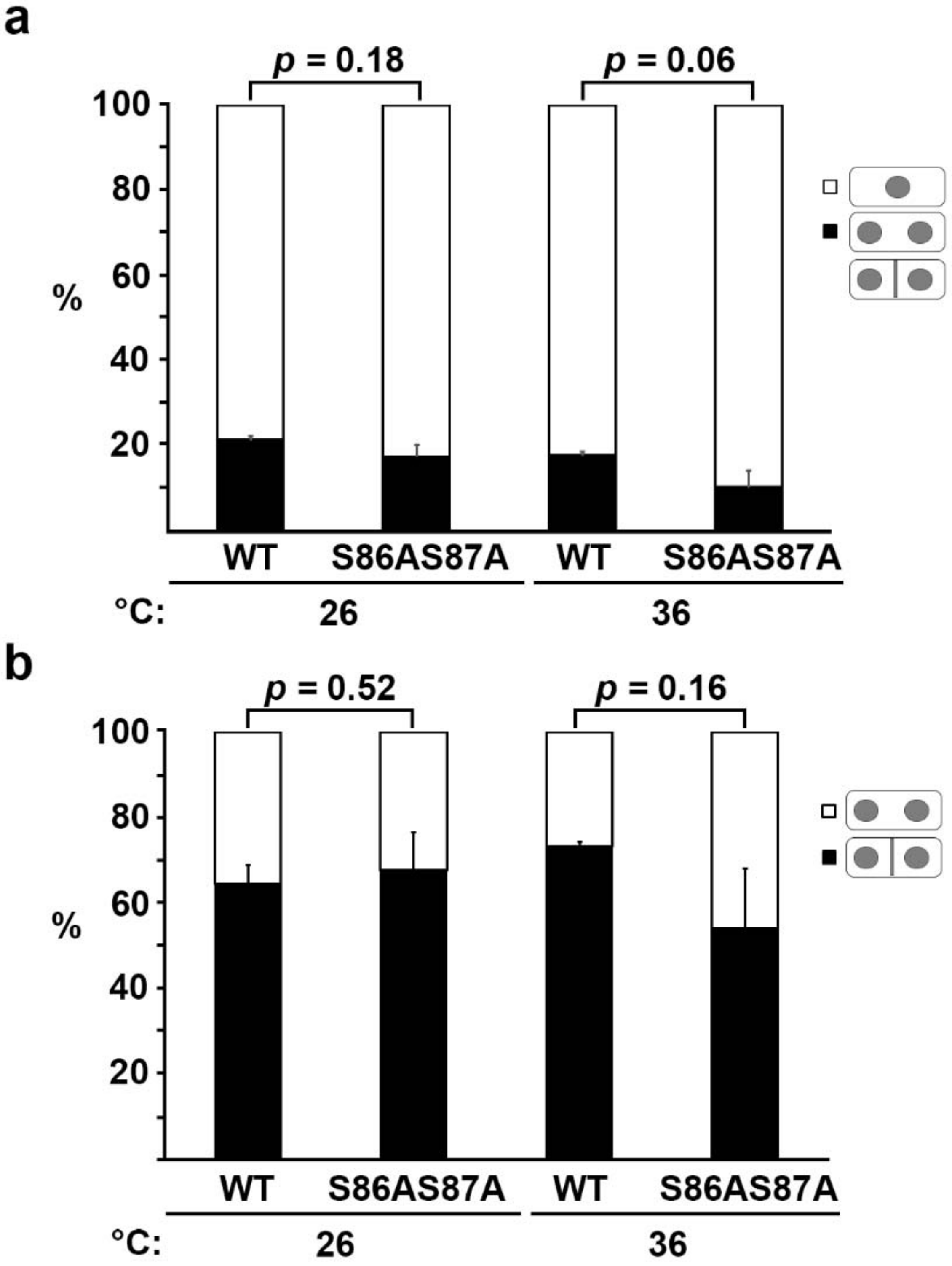

Supplementary Figure 6

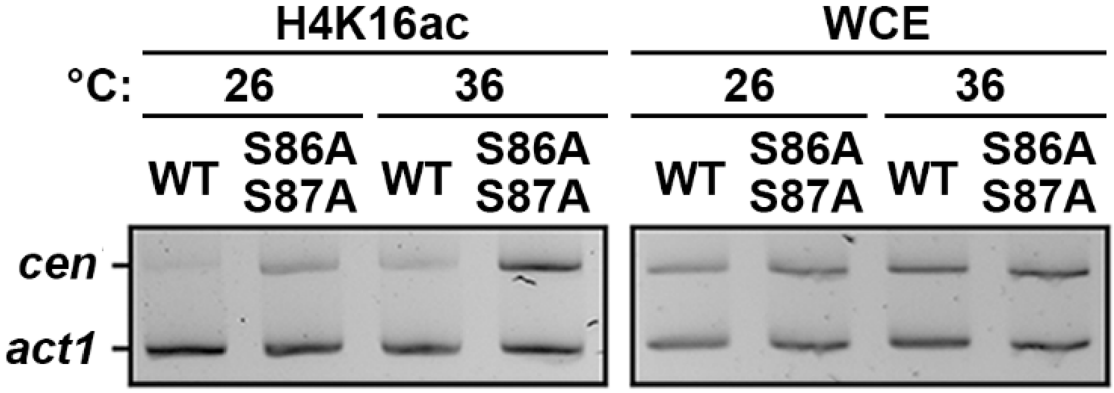

Supplementary Figure 7

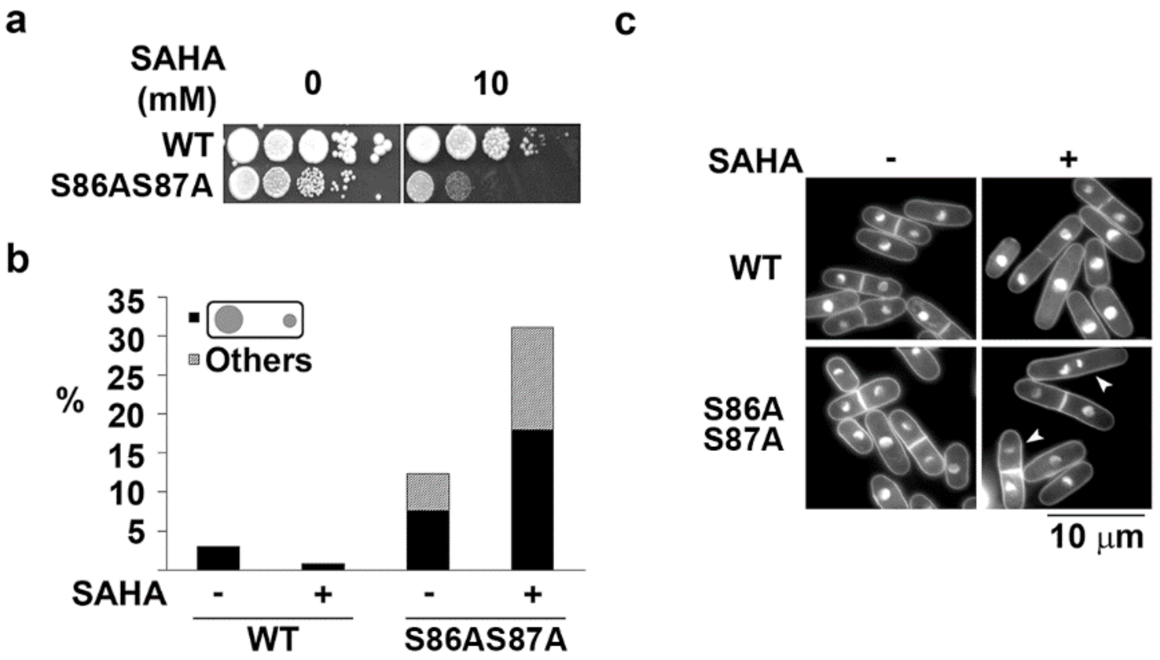

Supplementary Figure 8

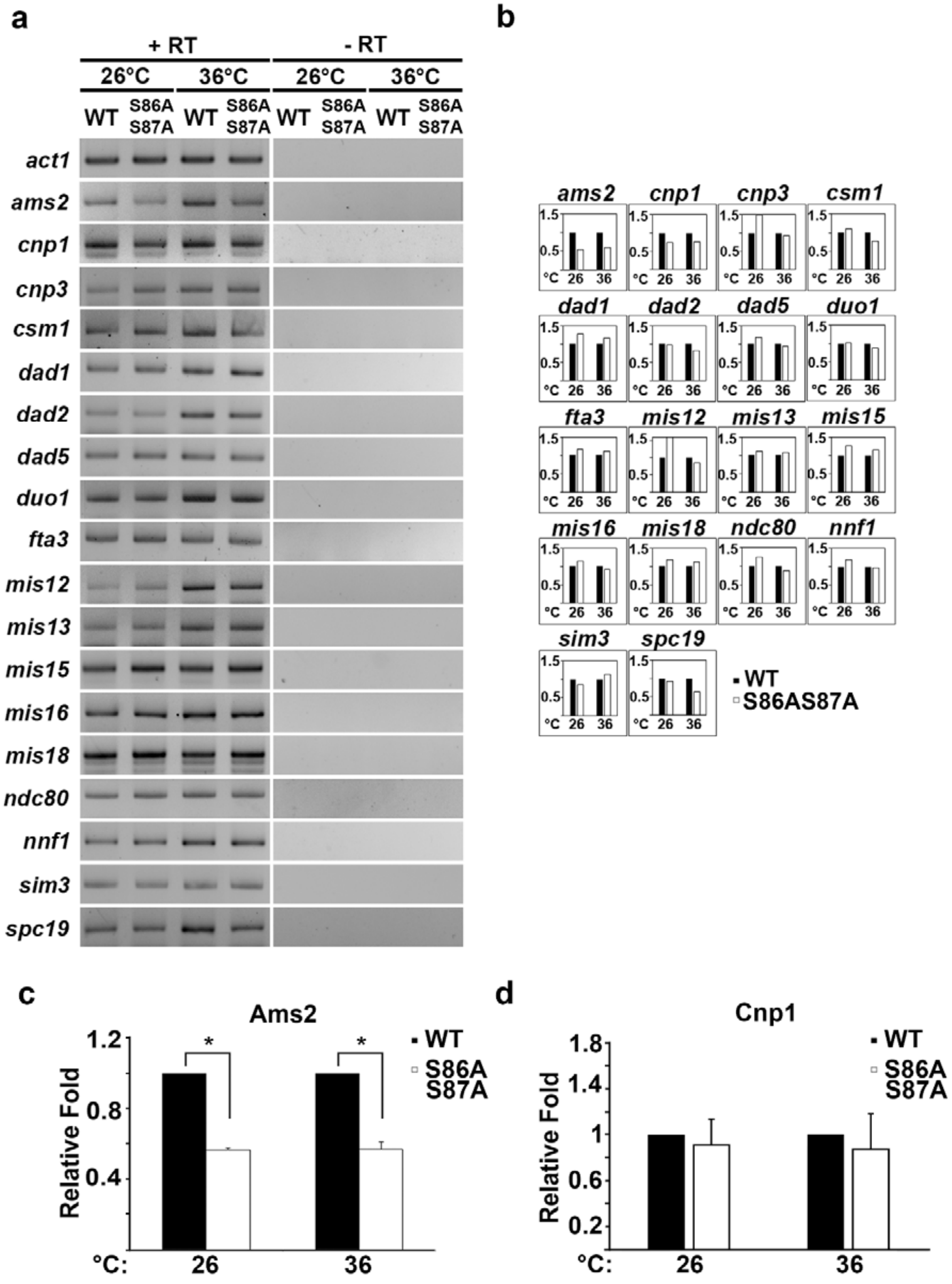

Supplementary Figure 9

a

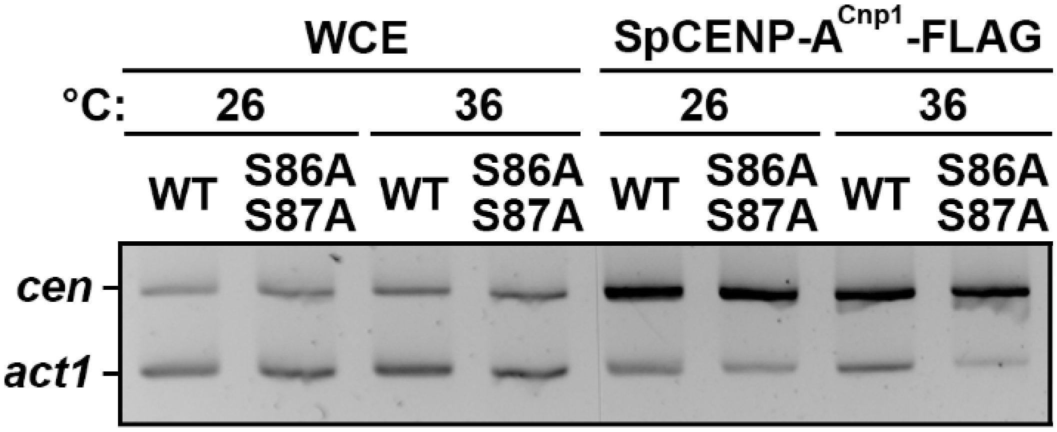

b

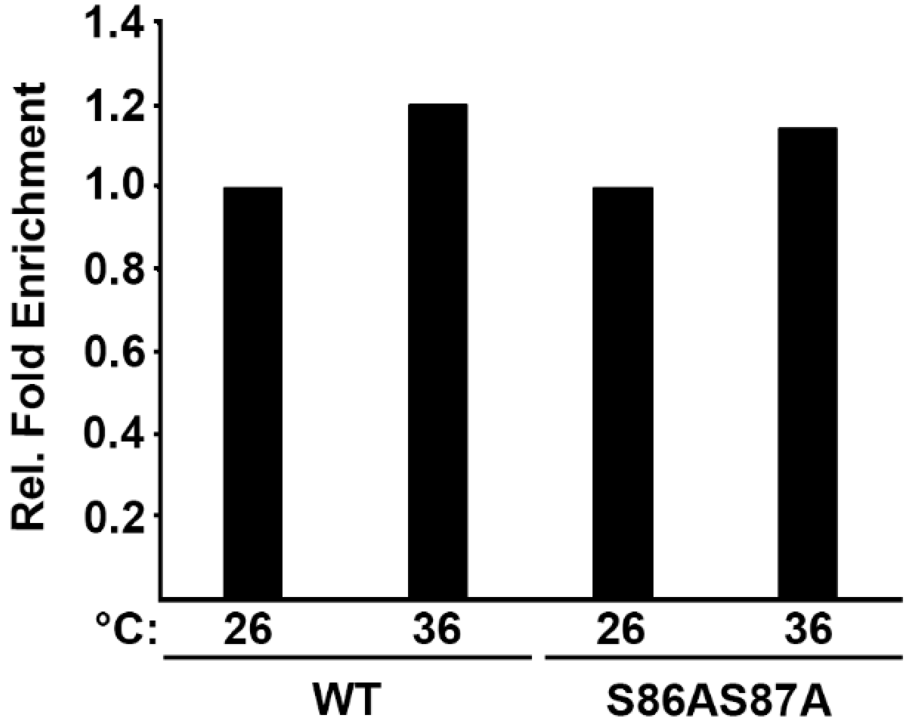

Supplementary Figure 10

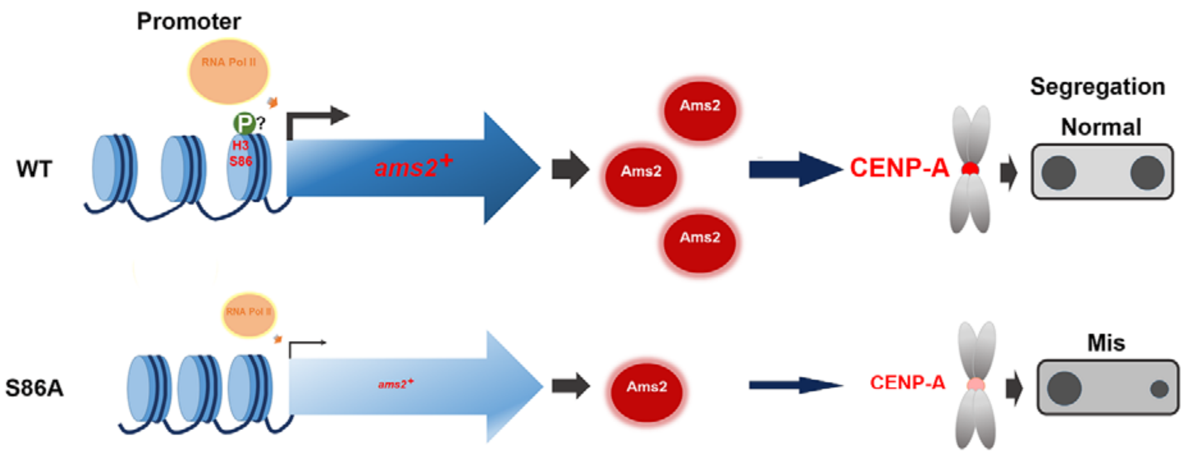

Supplementary Figure 11

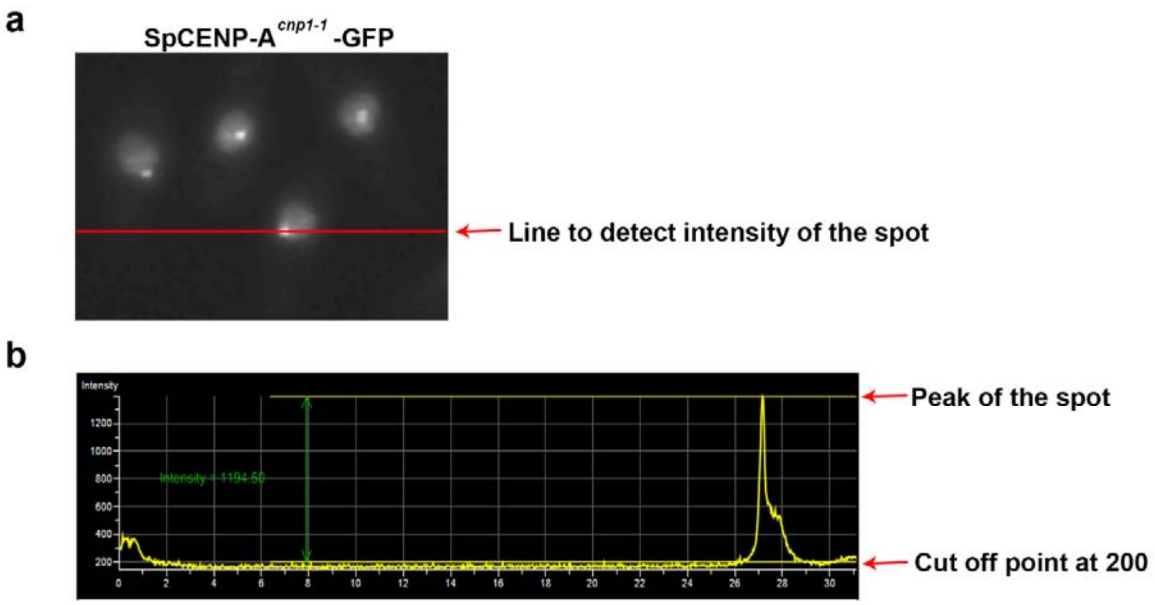

c

Intensity of SpCENP-A$^{cnp1\text{-}1}$-GFP spot = (Peak of the spot) - (Cut off point at 200)
